# Whole Transcriptome Analysis Provides Insights Into the Molecular Mechanisms of Chlamydospore-Like Cell Formation in *Phanerochaete chrysosporium*

**DOI:** 10.3389/fmicb.2020.527389

**Published:** 2020-12-07

**Authors:** Lei Liu, Huihui Li, Yanyan Liu, Yi Li, Hailei Wang

**Affiliations:** ^1^College of Life Sciences, Henan Normal University, Xinxiang, China; ^2^Advanced Environmental Biotechnology Center, Nanyang Environment and Water Research Institute, Nanyang Technological University, Singapore, Singapore

**Keywords:** *Phanerochaete chrysosporium*, chlamydospore, transcriptome (RNA-seq), *TEC1*, signaling pathways

## Abstract

*Phanerochaete chrysosporium* is a white rot fungus naturally isolated from hardwoods and widely used in environmental pollution control because it produces extracellular peroxidases. It forms chlamydospores during nitrogen starvation, which naturally occurs in the habitat of *P. chrysosporium*. Chlamydospores protect fungi against many stresses; the molecular basis underlying chlamydospore formation in basidiomycetes is poorly explored. Chlamydospores in *P. chrysosporium* have a different cell wall compared with hyphae, as confirmed by cell wall digestion and microscopy. Furthermore, this study investigated the transcriptome of *P. chrysosporium* in different life stages, including conidium, hypha, and chlamydospore formation, through RNA sequencing. A total of 2215 differentially expressed genes were identified during these processes. The expression patterns of genes involved in several molecular events critical for chlamydospore formation, including starch and sucrose metabolism, phosphatase and kinase, and transcription factors, were determined. This study serves as a basis for further investigating the function of chlamydospore formation in the biotechnologically relevant fungus *P. chrysosporium*.

## Introduction

*Phanerochaete chrysosporium* is a typical representative of white rot fungi belonging to Phanerochaetaceae under Polyporales, Agaricomycetidae, and Basidiomycota. This fungus has been extensively studied and used in environmental pollution control because of its ability to degrade a wide variety of non-phenolic and phenolic compounds by producing ligninolytic enzymes (Gao et al., 2010). *P. chrysosporium* can be identified by the morphological characteristics of membranaceous, crust-like fruiting bodies and the microscopic characteristics of conidial structure and hyphae. Thick-walled chlamydospores appear in the middle or at the end of hyphae, and they vary in size from 50 μm to 60 μm. Chlamydospores are morphological structures observed in fungi with enlarged, thick-walled vegetative cells and a condensed cytoplasm; this structure forms in the middle or top of the hyphae and can be used as a morphological identification of many fungal species (Barran et al., 1977; Ohara and Tsuge, 2004; Spraker et al., 2016). Environmental cues that trigger chlamydospore formation in fungi are usually species specific and include nutrients, osmolarity, light, pH, temperature, air, drug treatment, and plant stimulants.

Chlamydospores can be found in a number of fungi, including ascomycetes, saccharomycetes, and basidiomycetes. This life stage emerges under unfavorable conditions in some pathogenic fungi and promotes survival. Chlamydospores exhibit better stress resistance than conidia; as such, the former preserve, germinate, and survive easily (Jiménez-Tobon et al., 2003; Chung et al., 2005). Chlamydospores can be induced to form on hyphal tips via suspensor cells under nutrient-poor oxygen-limited conditions at low temperatures in *Candida albicans* (Calderone, 2002), whose natural habitats are humans and warm-blooded animals; this species has been widely studied because of its capacity to grow in several distinct morphological forms. Its chlamydospores are three to four times larger than those of yeast cells. In addition to *Candida* species, other fungal species belonging to *Cryptococcus*, *Trichoderma*, *Phytophthora*, *Fusarium*, *Ralstonia*, and *Clonostachys* genus can form chlamydospores (Lewis and Papavizas, 1984). Chlamydospores in *Cryptococcus neoformans* are rich in glycogen, suggesting that they play a role in energy storage (Lin and Heitman, 2005). Chlamydospores of *Trichoderma harzianum* and *Gliocladium roseum* can be induced by antifungal compounds produced by *Bacillus subtilis* (Li et al., 2005). Chlamydospores of *Phytophthora cinnamomi* are produced within plant roots during drought, are transported in root fragments or soil, and germinate to cause infections under warm and moist conditions (McCarren et al., 2005). Chlamydospores of *Fusarium* species promote long-term survival during unfavorable periods in soil, thereby resulting in the intercalary production of macroconidia and hyphae with a thickened wall or hyphal tips with a comparatively thin wall (Bandara and Wood, 1978). They play an important role as inoculum, with a greater potential of infecting plants than when compared to hyphae (Couteaudier and Alabouvette, 1990). Fusarium wilt, which is particularly difficult to control in strawberries, is also partly due to the ability of chlamydospores formed by pathogens to persist in soil for years (Cha et al., 2015). In another study, ralsolamycin, a diffusible metabolite produced by *Ralstonia solanacearum*, can facilitate its invasion of fungal hyphae. The fungi close to a *R. solanacearum* colony form distinct hyphal swellings that resemble chlamydospores, and the chlamydospores formed may provide a specific niche for bacterial colonization and enhance the survival of the symbiotic fungus (Spraker et al., 2016). Conversely, this structure can work as an antagonist that enables the biocontrol ability of fungi. The efficiency of the chlamydospores of *Clonostachys rosea* strain 67-1 to control the cucumber fusarium wilt exceeds 65% when chlamydospores are mixed with cucumber seeds at a concentration of 10^6^ spores/mL (Dong et al., 2014).

Chlamydospore formation in basidiomycetes has not been comprehensively studied. As one of the numbers of basidiomycetes, *P. chrysosporium* belongs to white rotting fungus and lives in rotted wood, which lacks nitrogen and experiences nitrogen starvation. Preliminary studies showed that a nitrogen-limited medium can induce chlamydospore formation in *P. chrysosporium* possibly to overcome this unfavorable environment. However, the genetic control of chlamydospore formation in *P. chrysosporium* remains to be studied. In the present study, RNA sequencing (RNA-seq) was conducted to investigate the changes in the expression of all genes involved in the chlamydospore formation of *P. chrysosporium* and to explore the mechanisms and molecular events associated with this process. RNA-seq is a high-throughput, deep-sequencing technology commonly used in genomic research to analyze the functional complexity of transcriptomes. It has a sufficient sensitivity and generates ultrahigh-throughput data, including several low-abundance genes; therefore, RNA-seq is more suitable and affordable for comparative gene expression than microarrays. Our findings provided important insights into the gene expression landscape of the chlamydospore formation of *P. chrysosporium* and provided a basis for identifying specific functional genes contributing to chlamydospore formation in other species.

## Materials and Methods

### Fungal Culture and Sample Preparation

*Phanerochaete chrysosporium* (ATCC24725) was obtained from the Henan Province Engineering Laboratory for Bioconversion Technology of Functional Microbes, China. It was incubated on potato dextrose agar (PDA) plates and subcultured for 3 days at 30°C. Subsequently, conidial suspensions (5 × 10^7^ spores/mL) were prepared in sterile water. For hyphal formation, the suspensions (2.0 mL/flask) were inoculated into 500-mL flasks containing 300 mL of PDA. The flasks were placed in a shaking incubator at 180 rpm and 37°C. For chlamydospore formation, the suspensions (5.0 mL/flask) were inoculated in 500-mL flasks containing a liquid medium (300 mL) composed of the following: 10.0 g/L glucose, 2.5 g/L (NH4)_2_SO_4_, 1.0 g/L KH_2_PO_4_, 0.8 g/L MgSO_4_, 0.5 g/L CaCl_2_ (Sigma-Aldrich, United States), and 0.7% trace element solution comprising 0.5 g/L glycine, 0.1 g/L FeSO_4_⋅7H_2_O, 0.1 g/L CoSO_4_, 0.1 g/L ZnSO_4_, 0.1 g/L MnSO_4_⋅H_2_O, 10 mg/L CuSO_4_⋅5H_2_O, 10 mg/L AlK(SO_4_)_2_⋅12H_2_O, 10 mg/L H_2_BO_3_, and 10 mg/L Na_2_MoO_2_⋅2H_2_O (Sangon Biotech, China). The sampling interval was 12 h, and the samples cultured for 36 and 72 h were taken out from the fermentation broth in a laminar flow bench after their morphological characteristics were observed under a microscope. The fungal biomass was freeze-dried before weighing. Cell wall thickness was determined via transmission electron microscopy (TEM) to measure the different parts of the cell wall with a scale mark from different hyphae and chlamydospores in accordance with a previously described protocol (Ferguson et al., 2005). Data were presented as mean ± standard error of the mean. The samples were washed twice with phosphate-buffered saline (PBS), stored, and then frozen in liquid nitrogen.

On the basis of preliminary experiments, the conidial structure (0 h, CK0) and the hyphae cultured in PDA at 36 h (CK36) and 72 h (CK72) were taken as the control group, while chlamydospore formation included two stages, namely, 36 h (T36) and 72 h (T72), were taken as the treatment group to understand the pattern of differential gene expression between the hyphae and the chlamydospores (Table 1). Then, 100 mg of the conidia, hyphae, and chlamydospores was separately added to 1.5-mL centrifuge tubes after 8000 r/min for 10 min and washed twice with PBS.

**TABLE 1:** Details of each sample.

**Sample**	**Time**	**Description**
CK0_1	0 h	Conidia
CK0_2	0 h	Biological replicate of CK0_1
CK36_1	36 h	36 h hyphae
CK36_2	36 h	Biological replicate of CK36_1
CK72_1	72 h	72 h hyphae
CK72_2	72 h	Biological replicate of CK72_1
T36_1	36 h	Chlamydospore starting produced from hyphae
T36_2	36 h	Biological replicate of T36_1
T72_1	72 h	Chlamydospores massively produced (>96%)
T72_2	72 h	Biological replicate of T72_1

Cell walls were stained with calcofluor white (Sigma-Aldrich, United States) at a final concentration of 1 mg/mL for 1 min before microscopy, and lipid bodies were stained by submerging the fungal sections in 10 μg/mL Nile red (Sigma-Aldrich, United States) for 5 min and then washed with 0.1 M PBS (Sangon Biotech, China). Coomassie brilliant blue (Njjcbio, China) was used to stain for 15 min to detect whether or not proteins were present in the cell wall. Excitation and emission filters were 350 and 400 nm, respectively. Images were observed under a fluorescence microscope (Leica DM2500, Germany) and a laser scanning confocal microscope (Zeiss LSM 800, Germany). The cell wall thickness of the chlamydospores was also measured using TEM (JEM-100CXII, Japan).

### Enzymatic Hydrolysis of Cell Wall Components

The chlamydospores were washed twice with PBS, acquired from the fermentation broth, and separated from the hyphae through an ultrasonic treatment for 20 min. They were pretreated by grinding in liquid nitrogen and then processed through Soxhlet extraction to eliminate interference from lipid bodies. The powders were ground in liquid nitrogen for 5 min, ultrasonically treated for 60 min, and then centrifuged at 10,000 rpm for 10 min to obtain a purified cell wall. The cell wall of the hypha was obtained via the same method. β-Glucanase powder (400 mg; Solarbio, China) was dissolved in 20 mL of sodium acetate buffer solution (pH 4.5) to obtain 20 mg/mL β-glucanase buffer with filtration. Samples were taken every 6 h for a total of 42 h for enzymatic hydrolysis by glucanase. Cellulase powder (320 mg; Sigma-Aldrich, United States) was dissolved in 20 mL of sodium acetate buffer solution (pH 4.5) to obtain 16 mg/mL cellulase buffer with filtration. Samples were collected every 6 h for a total of 30 h for enzymatic hydrolysis by cellulase. Chitinase powder (100 mg; Sigma-Aldrich, United States) was dissolved in phosphate buffer (pH 6.4) to obtain 20 mg/mL chitinase buffer with filtration. The sampling interval was 2 h for a total of 10 h for enzymatic hydrolysis by cellulase. The supernatant (100 μL) of enzymatic hydrolysis liquid was tested with the 3,5-dinitrosalicylic acid method to measure the resulting sugars (Frankeberger and Johanson, 1983).

### Preparation of the cDNA Library for RNA-Seq

All samples were immediately frozen in liquid nitrogen after preparation and then stored at −80°C until their RNA was isolated. Total RNA was extracted using a Trizol reagent in accordance with the manufacturer’s protocol (Invitrogen, China) and treated with DNase to remove DNA contamination. RNA degradation and contamination were monitored on 1% agarose gels. The purity of the RNA samples was checked with a NanoPhotometer spectrophotometer (Implen, CA, United States) at absorbance wavelengths of 260 and 280 nm. RNA concentration was measured with a Qubit RNA assay kit in a Qubit 2.0 fluorometer (Life Technologies, CA, United States), and RNA integrity was assessed using the RNA Nano 6000 assay kit of the Bioanalyzer 2100 system (Agilent Technologies, CA, United States). Exactly 3 μg of RNA per sample was used as the input material for the RNA sample preparations. Sequencing libraries were generated using a NEBNext Ultra RNA library prep kit for Illumina (NEB, United States) in accordance with the manufacturer’s recommendations, and index codes were added to the attribute sequences of each sample. mRNA was isolated and enriched from the total RNA by using oligo (dT) magnetic beads (Illumina, CA, United States). Then, mRNA was divided into short fragments to be used as templates for the random hexamer-primed synthesis of first-strand cDNA in a fragmentation buffer. The second-strand cDNA was synthesized using the buffer, deoxyribonucleotide triphosphate, RNase H, and DNA polymerase I. The remaining overhangs were converted into blunt ends via exonuclease/polymerase activities. The NEBNext Adaptor with a hairpin loop structure was ligated to prepare for hybridization after the adenylation of the 3′-ends of the DNA fragments. Library fragments were purified with an AMPure XP system (Beckman Coulter, Beverly, MA, United States) to select cDNA fragments with a length of 150–200 bp. Then, 3 μL of uracil-specific excision reagent enzyme (NEB, United States) was used with a size-selected adaptor-ligated cDNA at 37°C for 15 min, followed by 5 min at 95°C before quantitative real-time polymerase chain reaction (qRT-PCR). PCR was performed with Phusion High-Fidelity DNA polymerase, universal PCR primers, and the index (X) primer. The PCR products were purified (AMPure XP system), and the quality of the library was assessed on an Agilent Bioanalyzer 2100 system. The index-coded samples were clustered on a cBot Cluster Generation System by using the TruSeq PE Cluster Kit v3-cBot-HS (Illumina) in accordance with the manufacturer’s instructions. After cluster generation, the prepared library was sequenced on an Illumina Hiseq platform to generate 125 bp paired-end reads.

### Transcriptome Data Processing and Sequencing Data Assembly

Raw sequence data were transformed by base calling into sequence data and stored in FASTQ format. Raw reads were cleaned by removing adapter sequences, empty reads, and low-quality sequences and deposited in the NCBI Sequence Read Archive^1^ under the SRA accession number SRP153122. The clean reads generated through RNA sequencing were mapped to the reference genome of *P. carnosa*, which has a close phylogenetic relationship to *P. chrysosporium*. An index of the reference genome was built using Bowtie v2.2.3 (Langmead and Salzberg, 2012), and paired-end clean reads were aligned to the reference genome via TopHat v2.0.12 (Trapnell et al., 2009). TopHat was selected as the mapping tool because it can generate a database of splice junctions based on the gene model annotation file and produce better mapping results than other non-splice mapping tools.

### Normalized Expression Levels of Genes From RNA-Seq

The gene expression levels based on the read counts obtained via HTSeq v0.6.1 were normalized through transformations with fragments per kilo base per million mapped reads (FPKM) to eliminate the influence of different gene lengths and sequence discrepancies on expression calculations (Anders et al., 2015). Transcripts with FPKM < 1 were filtered out, and the filtered transcripts were used as a reference for downstream analysis (Huang et al., 2015). The calculated gene expression levels could be used for the direct comparison of the samples. Expression values were standardized across the dataset to combine the data from different genes.

### Analysis of Differentially Expressed Genes (DEGs), Gene Ontology (GO), and Kyoto Encyclopedia of Genes and Genomes (KEGG)

The two conditions or groups (two biological replicates per condition) were subjected to differential expression analysis by using the DESeq R package (1.18.0; Wang et al., 2010). DESeq R provides statistical functions to determine differential expression in digital gene expression data via a model based on the negative binomial distribution. The resulting p-values were adjusted with the Benjamini–Hochberg method to control the false discovery rate (FDR) via the sequentially modified Bonferroni correction for testing multiple hypotheses (Benjamini and Hochberg, 1995). Positive and negative log2FoldChange (log2FC) values indicated significantly upregulated and downregulated genes, respectively. | log 2 FC| > 1 and FDR < 0.01 were considered as the cutoff values for DEG screening. The genes screened were subjected to GO enrichment analysis in GOseq R package. In this method, the gene length bias was corrected. The GO terms with the corrected *p* < 0.05 were considered significantly enriched by DEGs. The KEGG database was used to assign and predict the putative functions and pathways associated with the assembled sequences (Kanehisa and Goto, 2000). KOBAS software was utilized to test the statistical enrichment of DEGs in the KEGG pathways (Mao et al., 2005).

### RT-qPCR

RNA was isolated using the Ezgene^TM^ Fungal RNA Miniprep kit (Biomiga, San Diego, CA, United States). In this procedure, 50 ng of RNA was transcribed to cDNA by using Omniscript RT kit (Qiagen, Hilden, Germany) in accordance with the manufacturer’s instructions. Six genes were chosen to validate the RNA-seq differential gene expression data via qRT-PCR. The primers were designed with Primer version 5.0 based on the assembled transcriptome used for amplification. The product annotations are listed in Supplementary Table S1. β-Actin was used as an internal reference gene, and the relative gene expression levels were calculated using the comparative Ct method with the formula 2^–ΔΔCt^ (Livak and Schmittgen, 2001). RT-qPCR analyses were run in triplicate with three biological replicates. Then, RT-qPCR results were compared with the transcriptome data to detect the correlation of each gene expression.

### Statistical Analysis

Statistical analysis was conducted in GraphPad Prism 8 (GraphPad Software Inc., San Diego, CA, United States) for Windows. The means ± standard error of the mean (SEM) were used between the treatments. A *t*-test was performed to evaluate the statistical significance of independent groups. The correlation coefficient (*r*) between RNA-Seq and qPCR was calculated using two-tailed *p*-value with a confidence interval of 95%.

## Results

### Morphological Characteristics of the Conidia, Hyphae, and Chlamydospores

**FIGURE 1:**
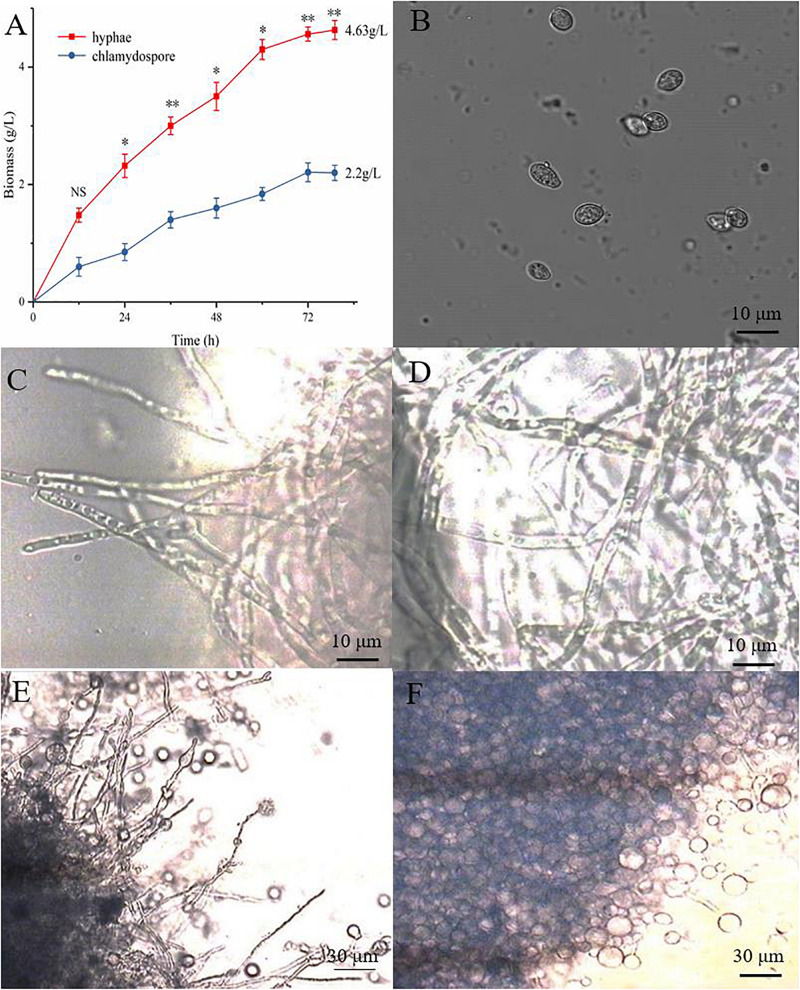
Different growth statuses of the hyphae and chlamydospores of *P. chrysosporium* under a light microscope. **(A)** The biomass of hyphae and chlamydospores cultured from 0 h to 72 h. **(B)** *P. chrysosporium* conidia (CK0). **(C)** Hyphae cultured for 36 h (CK36). **(D)** Hyphae cultured for 72 h (CK72). **(E)** Chlamydospore cultured for 36 h (T36) and **(F)** chlamydospore cultured for 72 h (T72). (Student’s *t*-test. Asterisks represent significant differences, **p < 0.01 and *p < 0.05).

**FIGURE 2:**
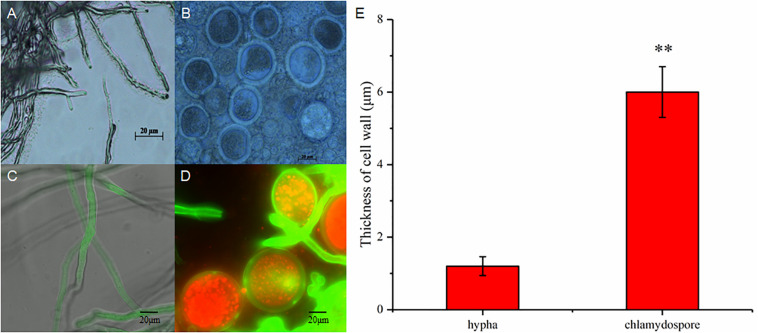
Different morphological characteristics of hyphae and chlamydospores. **(A)** Light microscopy of the hyphae at 72 h. **(B)** Light microscopy of the chlamydospores at 72 h. **(C)** Laser scanning confocal microscope (LSCM) of the hyphae at 72 h. **(D)** LSCM of the chlamydospores at 72 h. **(E)** measurement of the thickness of the cell wall between the hyphae and the chlamydospores. Asterisk indicates that the thickness of the cell wall was significantly different between hyphae and chlamydospores (Student’s *t*-test, **p < 0.01).

The hyphae and the chlamydospores were observed and recorded in different culture phases to investigate the phenotypic changes between them. The maximum biomass of the hyphae and the chlamydospore reached 4.63 and 2.2 g/L, respectively, after cultivation for 72 h (Figure 1A). Three types of cells, namely, CK0 (conidia, Figure 1B), CK36 (hyphae, 36 h growth, Figure 1C), CK72 (hyphae, 72 h growth, Figure 1D), T36 (chlamydospores, 36 h, Figure 1E), and T72 (chlamydospores, 72 h, Figure 1F) were analyzed via light microscopy. The remarkable characteristics of the thickened cell wall were observed for the chlamydospores in comparison with the hyphae (Figures 2A–D). The thickness of the cell wall increased with prolonged culture time, and the overall thickness of the entire wall of 60 hyphae and mature chlamydospores was measured. The maximum thickness of the cell wall of the chlamydospores reached 6.7 μm (Figure 2E). Optical microscopy confirmed that the cell walls of the cultured chlamydospores (Figure 2B) were thicker than those of the hyphae (Figure 2A). After the samples were stained with calcofluor white, laser confocal scanning microscopy revealed that the fluorescence intensities of the cell wall of the chlamydospores (Figure 2D) were stronger than those of the cell wall of the hyphae (Figure 2C). Nile red staining also showed that the chlamydospores contained large lipid bodies (Figure 2D). The thickened part of the cell wall was not stained with Coomassie brilliant blue, indicating that this part rarely contained or did not have proteins (Supplementary Figure S1).

### Enzymatic Hydrolysis by Glucanase, Cellulase, and Chitinase

**FIGURE 3:**
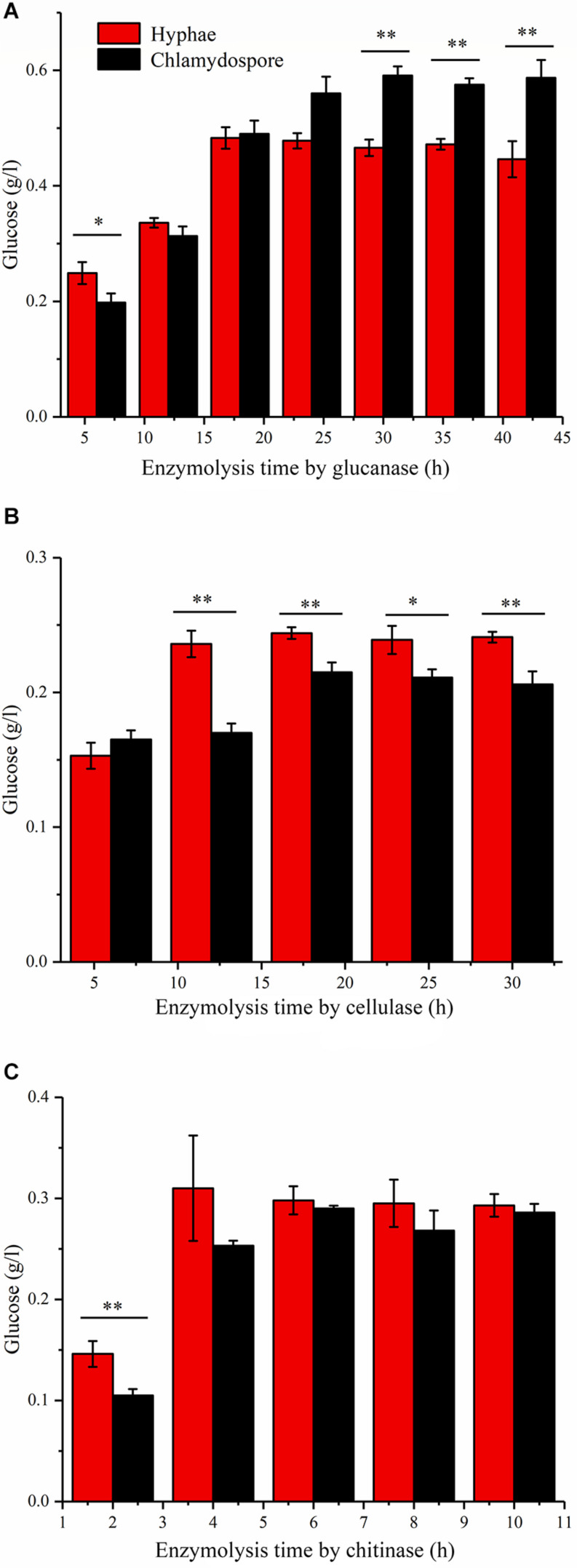
Analysis of the polysaccharide composition of the cell wall of the hyphae and chlamydospores through the enzymatic hydrolysis of the extract. **(A)** Enzymatic hydrolysis by glucanase. **(B)** Enzymatic hydrolysis by cellulase. **(C)** Enzymatic hydrolysis by chitinase. All data are presented as the means ± standard error and evaluated using Student’s *t*-test. Asterisks represent significant differences (**p < 0.01 and *p < 0.05).

Enzymatic hydrolysis by glucanase, cellulose, and chitinase was experimentally studied to further understand the different compositions of polysaccharides between the hyphae and the chlamydospores. β-Glucans are natural polysaccharides with a molecular weight of over 6400 daltons and composed of β-(1-3)-glucans, β-(1-4)-glucans, or β-(1-6)-glucans. β-Glucanase can specifically hydrolyze glucan into glucose as the final enzymatic hydrolysate. As such, the reducing sugar in the hydrolysate of 20 mg of the cell wall of the chlamydospores was tested after β-glucanase treatment. An equivalent amount of the cell wall of the hyphae was used as the control. Before the experiment, the reducing sugar content was tested, and the result indicated that neither the samples nor the enzymes contained a reducing sugar before processing (Figure 3A). In the first 18 h of enzymatic hydrolysis, the reducing sugar content in the enzymatic hydrolysates of the hyphae and the chlamydospores increased. The reducing sugar content in the hyphal enzymatic hydrolysate was slightly higher than that in the chlamydospore enzymatic hydrolysate. After 18 h, the reducing sugar content in the hyphal enzymatic hydrolysate no longer changed, and the maximum content was 0.480 mg/mL. However, the reducing sugar content detected in the chlamydospore enzymatic hydrolysate continued to increase and reached the peak concentration of 0.598 mg/mL at 30 h (Figure 3A). This phenomenon may be attributed to the relatively dense thickened part of the chlamydospore cell wall, thereby causing low-efficiency catalysis. Glucan in the cell wall was gradually digested by the enzyme as enzymatic hydrolysis continued. The results indicated that the glucan content in the chlamydospore cell wall was higher than that in the hyphal cell wall at equal mass. However, it might have also resulted from the different organizations of the polysaccharides of the cell walls of the chlamydospores and the hyphae. Consequently, more β-glucans were exposed to β-glucanase in the chlamydospores.

Cellulose is another polysaccharide and important component of the fungal cell wall. It can be degraded generally by cellulase to generate glucose. The cellulose contents in the substrate of the chlamydospores and the hyphae can be determined by measuring and comparing the glucose contents before and after enzymatic hydrolysis. In this study, 20 mg of hyphae and 20 mg of chlamydospores were used in enzymatic hydrolysis (Figure 3B). The degradation rate of cellulose in the chlamydospore cell wall was high, and its glucose content was slightly higher than that in the hyphal cell wall in the first 6 h. Nevertheless, the enzymatic hydrolysis of the chlamydospore group tended to be stable, and the hydrolysate content reached the maximum of 0.215 mg/mL at 18 h. Similarly, the hydrolysate content in the hyphal group reached the maximum of 0.244 mg/mL at 18 h. Subsequently, the glucose content was maintained at a stable level. The glucose content in the hyphal group remained higher than that in the chlamydospore group from 6 h to the end of the experimental period. The cellulose concentration in the chlamydospore cell wall was lower than that in the hyphal cell wall. However, the organization of cell wall polysaccharides of the chlamydospores might differ from that of the hyphae likely because the amount of cellulose exposed to cellulase in the chlamydospores was lower than that in the hyphae.

Chitin is a straight-chain macromolecule composed of *N*-acetylglucosamine residues linked by β-1,4-glycosidic bonds. It widely exists in crustaceans and in the cell wall of filamentous fungi and some green algae. In this experiment, 5 mg of hyphal cell wall was digested by the enzyme at an equal rate and reached the maximum chitin concentration of 0.313 mg/mL in the first 4 h of enzymatic hydrolysis. The chitinase hydrolysis rate of the chlamydospore cell wall with the same quantity was lower than that of the hyphal cell wall at the initial stage. Afterward, it increased gradually possibly because of the weak combination between the enzyme and the thick dense cell wall of the chlamydospores. The enzymatic hydrolysis of the hyphae stopped after 6 h, whereas the enzymatic hydrolysis of the chlamydospores continued at a slow rate at 6 h and stopped at 8 h (Figure 3C). The enzymatic hydrolysis of the chlamydospores yielded the maximum chitin concentration of 0.290 mg/mL. Therefore, the chitin contents in the cell walls of the hyphae and the chlamydospores were basically consistent. Consequently, the results of all above enzymatic hydrolysis supported the microscopic observation and suggested significant differences in cell wall structure and organization between the chlamydospores and the hyphae in *P. chrysosporium*.

### RNA-Seq and DEGs (Differentially Expressed Genes) Analyses at Different Stages

The hyphae and the chlamydospores at different stages were subjected to RNA-seq analysis in order to identify the transcriptional patterns associated with chlamydospore development. The sequencing results are summarized in Table 2. The proportion of the clean reads to the raw reads in the 10 cDNA libraries ranged from 96.06% to 96.67%. Overall, 76.68% to 80.96% of the clean reads were aligned against the *P. carnosa* genome, and the uniquely mapped reads ranged from 76.23% to 80.44%. The average guanine–cytosine content of the clean reads from the 10 libraries was 59.82. The proportion of the reads with the Phred quality value of Q20 among the clean reads ranged from 97.46% to 97.82%, and the Q30 among the clean reads ranged from 94.02% to 94.58%. The error rate of a single base was 0.01%.

**TABLE 2:** The quality of transcriptome sequencing data.

**Sample name**	**Raw reads**	**Clean reads**	**Clean base (G)**	**Error rate (%)**	**Q20 (%)**	**Q30 (%)**	**GC (%)**
CK0_1	35441486	34065626	4.2	0.01	97.46	94.02	59.94
CK0_2	36137676	34880668	4.36	0.01	97.68	94.40	60.00
CK36_1	30940888	29733958	3.72	0.01	97.54	94.15	60.36
CK36_2	28013254	26973044	3.37	0.01	97.67	94.36	60.06
CK72_1	29965294	28968620	3.62	0.01	97.82	94.58	59.49
CK72_2	32299912	31179170	3.90	0.01	97.72	94.39	59.82
T36_1	37415538	36022412	4.50	0.01	97.72	94.48	59.99
T36_2	20588054	29565202	3.70	0.01	97.73	94.40	58.59
T72_1	33169438	31960838	4.00	0.01	97.54	94.09	60.11
T72_2	33871300	32666572	4.08	0.01	97.72	94.45	59.85

**FIGURE 4:**
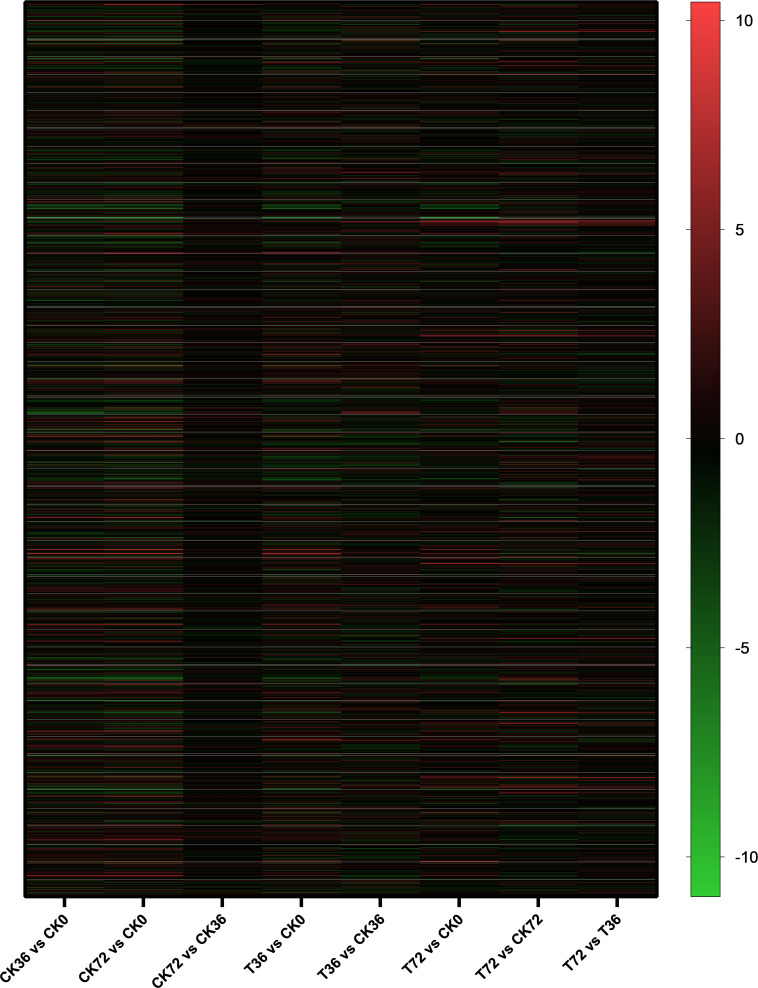
Hierarchical cluster analysis of the gene expression based on the log ratio RPKM data of *P. chrysosporium* in terms of its chlamydospore formation. Red represented a relatively high expression level, and blue corresponded to a relatively low expression level. Each column showed an experimental condition (CK0-T72), and each row indicated a gene.

**FIGURE 5:**
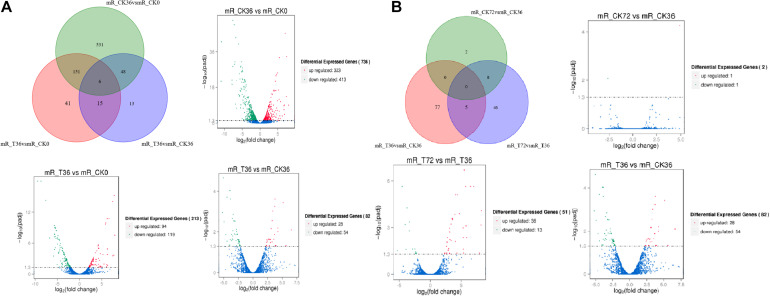
Venn chart of unique and common differentially expressed genes (DEGs) between different stages of chlamydospore formation. **(A)** The grouping of the gene lists responding to T36 vs. CK36 vs. CK0. **(B)** The grouping of the gene lists responding to T72 vs. CK72 vs. T36 vs. CK36.

The clean reads were assembled to understand the DEGs associated with chlamydospore formation. The results revealed 32,016 genes, which were used in sequential DEG analyses. Subsequently, a global view of the gene expression pattern of DEGs was generated through hierarchical cluster analysis (Figure 4). In the first 36 h, 805 differentially expressed mRNAs were obtained through the pairwise comparison of T36 vs. CK36 vs. CK0. Results showed the following: 736 DEGs between the Ck36 and Ck0 groups, i.e., 323 upregulated genes and 413 downregulated genes; 213 DEGs between the T36 and CK0 groups, i.e., 94 upregulated genes and 119 downregulated genes; and 82 DEGs between T36 and CK36, i.e., 28 upregulated genes and 54 downregulated genes (Figure 5A). From 36 h to 72 h, only 130 differentially expressed mRNAs were obtained after the three groups were compared (T72 vs. CK72 vs. T36 vs. CK36). Furthermore, two DEGs, namely, one upregulated gene and one downregulated gene, were found between the CK72 and CK36 groups, and 51 DEGs, i.e., 38 upregulated genes and 13 downregulated genes, were observed between the T72 and T36 groups (Figure 5B). These results indicated the difference in the gene expression levels between the samples.

### Formation of Relevant Differential Gene KEGG (Kyoto Encyclopedia of Genes and Genomes) Enrichment Pathways With Chlamydospores

**FIGURE 6:**
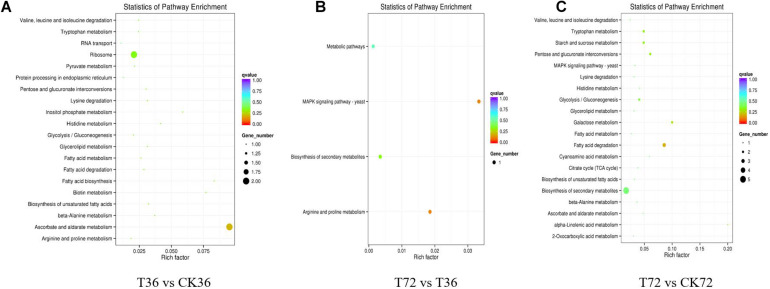
KEGG pathway enrichment analysis of the differentially expressed genes (DEGs) of chlamydospore formation. **(A)** T36 vs. CK36, **(B)** T72 vs. T36, and **(C)** T72 vs. CK72.

The DEGs were searched against the KEGG database to understand the potential molecular pathways underlying chlamydospore formation. Chlamydospore formation was related to the regulation of biomacromolecule synthesis and decomposition, which involved carbohydrate, lipid, and protein metabolism. Comparison of the enrichments revealed that most of the DEGs were involved in various pathways (Table 3), including ribosome, ascorbate, and aldarate metabolism in T36 vs. CK36 (Figure 6A); secondary metabolite biosynthesis, arginine and proline metabolism, MAPK signaling pathway, and metabolic pathways in T72 vs. T36 (Figure 6B); and secondary metabolite biosynthesis, fatty acid degradation, starch and sucrose metabolism, glycolysis/gluconeogenesis, galactose metabolism, tryptophan metabolism, and pentose and glucuronate interconversions in T72 vs. CK72 (Figure 6C). These pathways indicated that the large-scale assembly of carbohydrates might participate in chlamydospore formation.

**TABLE 3:** Significantly changed Kyoto Encyclopedia of Genes and Genomes (KEGG) pathways.

**Developmental stages**	**Pathway**	***P*-value (<0.05)**	**Gene name**	**Blast swiss prot**	**Up/Down**
T36 vs. CK0	Galactose metabolism	0.0197392327084	Unknown	UTP–glucose-1-phosphate uridylyltransferase	Up
			Unknown	Aldose 1-epimerase	Up
	Fatty acid degradation	0.0247110569989	Unknown	Alcohol dehydrogenase, propanol-preferring	Down
			Unknown	Cytochrome P450/NADPH-cytochrome P450 reductase	Down
T36 vs. CK36	Ribosome	0.00317042906325	S15e	Small subunit ribosomal protein S15e	Up
			LP1, LP2	Large subunit ribosomal protein LP1, LP2	Up
	Ascorbate and aldarate metabolism	0.00621469206944	Unknown	Inositol oxygenase	Down
			Unknown	Aldehyde dehydrogenase family 7 member A1	Down
T72 vs. CK0	Fatty acid degradation	0.0230827057547	Unknown	Alcohol dehydrogenase, propanol-preferring	Down
			Unknown	Cytochrome P450/NADPH-cytochrome P450 reductase	Down
T72 vs. CK72	Fatty acid degradation	0.0298625776715	Unknown	Acyl-CoA oxidase	Up
			Unknown	Aldehyde dehydrogenase family 7 member A1	Up
	Alpha-Linolenic acid metabolism	0.043461306319	ACX	Acyl-CoA oxidase	Up
	Starch and sucrose metabolism	0.00637655688167	Unknown	Beta-glucosidase	Down
			Unknown	UTP–glucose-1-phosphate uridylyltransferase	Down
T72 vs. T36	MAPK signaling pathway - yeast	0.0293679603427	Tec1	Transcriptional enhancer factor	Up

**FIGURE 7:**
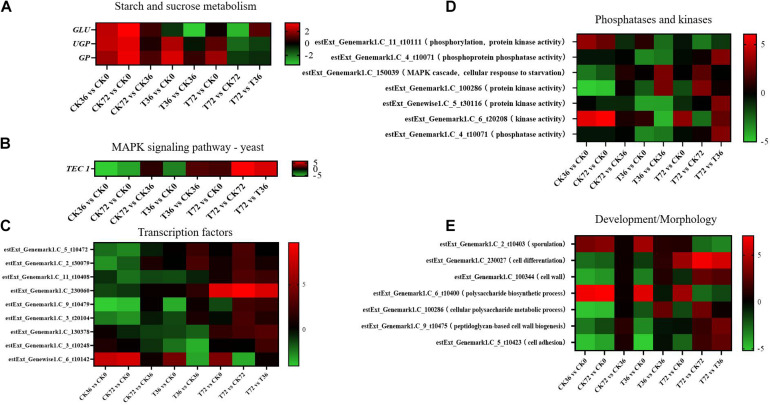
Heatmap of the genes regulated at different stages of chlamydospore formation. The bar represents log2 (fold change) between different groups. Red represents upregulation, and green shows downregulation. **(A)** *GLU*, *UGP*, and *GP*. **(B)** *TEC* 1. **(C)** Transcription factor genes. **(D)** phosphatase and kinase genes. **(E)** Development/morphology genes.

Two sugar metabolism-related genes were differentially regulated in *P. chrysosporium*. Uridine triphosphate (UTP)–glucose-1-phosphate uridylyltransferase gene (*UGP*; log2FoldChange = −1.8348) and β-glucosidase gene (*GLU*; log2FoldChange = −3.2038) were greatly downregulated in the starch and sugar metabolism of the enrichment pathways in the T72 vs. CK72 groups (Figure 7A). *UGP*, also known as glucose-1-phosphate uridylyltransferase or uridine diphosphate (UDP)–glucose pyrophosphorylase, is an enzyme involved in carbohydrate metabolism. *UGP* synthesizes UDP–glucose from glucose-1-phosphate and UTP. The downregulation of this gene might cause the partition of the sugar flux toward cell wall synthesis, including cellulose and 1,4-β-D-glucan syntheses. *GLU* belongs to glycoside hydrolase family 1, which encodes a wide range of enzymes that hydrolyze the glycosidic bond between two or more carbohydrates or between a carbohydrate and a non-carbohydrate moiety. *GLU* expression can catalyze the hydrolysis of 1,4-β-D-glucan into β-D-glucose. The downregulation of this gene in T72 might result in the accumulation of 1,4-β-D-glucan in cells (Figure 7A). The glycogen phosphorylase gene (*GP*), which helps remove glucose from glycogen, can produce glucose-1-phosphate, which can be used to produce ATP. However, it was significantly regulated only during the hyphal growth (CK36 vs. CK0, CK72 vs. CK0) and the initial stage of chlamydospore formation (T36 vs. CK0). Therefore, *GP* might not play a key role in chlamydospore formation (Figure 7A).

Mitogen-activated protein kinase (MAPK) pathways control the reproduction, spore morphology, and expression of the pathogenic toxicity factor and is vital in the synthesis and integrity of the fungal cell wall (Mehrabi et al., 2006). In *Schizosaccharomyces pombe*, the loss of any of the kinases in this pathway results in the appearance of rounded cells instead of rod-like cells that are sensitive to β-glucanase; this finding suggests that this pathway is involved in cell wall maintenance (Nobel et al., 2000). MAPKs coordinate and execute cellular responses to environmental signals (Pearson et al., 2011). External stimuli, such as oxygen pressure, hunger, osmotic pressure, heavy metals, and low temperature, can activate MAPK signaling pathways. Most fungi always have four major MAPK pathways, including the Fus3/Kss1, Kss1, Slt2, and Hog1 pathways. In the pheromone response pathway, Fus3 and Kss1 MAPKs regulate the mating processes. For filamentous growth pathways, KSS1 is the final kinase in the signal transduction cascade regulating activation/repression of the filamentation induced by nitrogen/carbon limitation. The Slt2 cell integrity pathway monitors cell wall integrity and promotes cell wall biosynthesis, and the high-osmolarity glycerol (Hog 1) response pathway is required for growth under hyperosmotic conditions (Zhao et al., 2007). The synthesis of chlamydospores started from 36 h, and hyphae were basically transformed into chlamydospores at 72 h. According to the enrichment of the T72 vs. T36 groups, the Kss1 (starvation) pathway showed a remarkable enrichment that plays an important role in cell cycle regulation. The transcriptional enhancer factor Tec1 functions by assembling with another transcription factor Ste12 in the Kss1 pathway in yeast (Shock et al., 2009). Ste12 and Tec1 factors activate genes involved in biofilm/filamentation programs (van der Felden et al., 2014). Ste12–Tec1 heterodimers bind cooperatively to a distinct DNA sequence called the filamentation and invasion response element present in the promoter region of genes that regulate invasive or pseudohyphal growth (Wang and Dohlman, 2006). *TEC1* was substantially upregulated in the targets of the T72 vs. T36 groups (log2FoldChange = 5.3222) and conserved with Ste12, which might promote hyphal growth (Figure 7B). *P. chrysosporium* chlamydospores were produced primarily within the hyphal cells or at the end of hyphae, and upregulating *TEC1* might positively promote hyphal elongation, which induced the formation of additional chlamydospores. *TEC1* in *P. chrysosporium* had low amino acid sequence identity (27.94%, e-value = 4e − 06) compared with *Saccharomyces cerevisiae*, which may indicate divergence of function. Moreover, it has not been studied in other species closely related to *P. chrysosporium*. Thus, its significance and role in basidiomycete chlamydospore formation remain enigmatic but present an interesting target for studies aiming to understand chlamydospore formation in this fungus.

Other genes, such as those encoding transcription factors, phosphatases, and kinases, and development/morphology-related components, may play important roles in chlamydospore formation. Functional annotation revealed the differential expression of eight transcription factors (Figure 7C), seven phosphatase and kinase genes (Figure 7D), and seven genes involved in development/morphology (Figure 7E). Most transcription factor, phosphatase, and kinase genes were significantly upregulated during chlamydospore production (T72 vs. T36), but these genes remain uncharacterized in fungi to date. Further verification through deletion of these genes is needed to clarify their functions in chlamydospore formation.

**FIGURE 8:**
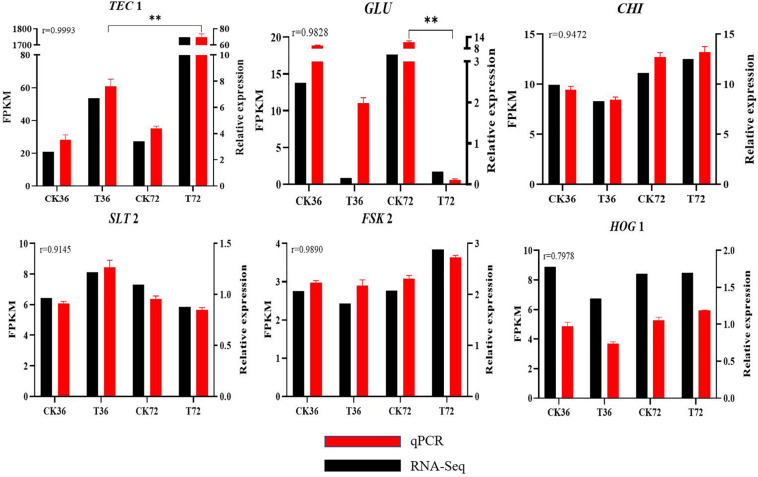
Real-time qPCR validation showing the relative expression levels of *GLU*, *TEC* 1, *CHI*, *SLT* 2, *FKS* 2, and *HOG* 1. (Student’s *t*-test. Asterisks represent significant differences, **p < 0.01).

qRT-PCR results are shown in Supplementary Figure S2 to validate the reliability of DEGs obtained from RNA-seq analysis. r was calculated between the RNA-seq and qRT-PCR data of six genes. The expression patterns of the six genes determined through qRT-PCR were consistent with the RNA-seq data, indicating the reliability and accuracy of the RNA-seq data (Figure 8).

## Discussion

Early research found that the cell wall of *Aspergillus flavus* chlamydospores could stain calcofluor white, which demonstrated that chlamydospore cell walls are thicker than normal hyphal cell walls (Spraker et al., 2016). In *C. neoformans*, the diameter of chlamydospores is greater than 10 μm, which is significantly larger than the almost uniform 2–5 μm round blastospores or 1.5–3 μm oval basidiospores (Lin and Heitman, 2005). In the current work, hypertrophic structures in thick-walled chlamydospores were observed by cell wall-specific staining and demonstrated that chlamydospore cell walls are thicker than normal hyphal cell walls. Enzymatic hydrolysis also suggested the differences in polysaccharide compositions of the cell walls between chlamydospores and hyphae. Some essential genes for chlamydospore formation have been determined through specific mutation in *C. albicans* (Staib and Morschhäuser, 2007; Böttcher et al., 2016; Noble et al., 2016) and *Gibberella zeae* (Son et al., 2012), but genes in the chlamydospore formation of *P. chrysosporium* have not been investigated. Efg1p, which is the first discovered regulatory factor and encoded by *EFG* for chlamydospore formation in *C. albicans*, can regulate morphogenesis and induce mycelial growth (Stoldt et al., 1997). *HOG1* in the MAPK pathway is another gene related to the formation of chlamydospores in *C. albicans* (Alonso-Monge et al., 2003). *C. albicans HOG1* is activated in response to hydrogen peroxide and an increase in external osmolarity. Chlamydospores, which are round and have thick walls, have been observed in wild-type *HOG1*, whereas chlamydospore formation is completely abolished in *HOG1* mutants (Staib and Morschhäuser, 2007). The colonies display the hyperfilamentous phenotype of *HOG1* mutants, and some structures at some hyphal tips slightly resemble chlamydospores (Alonso-Monge et al., 2003). *Ole1* is not universal in filamentous fungi, but it may be an important inducer of some signaling pathways for specialized developmental structures. *Ole1* can make saturated fatty acids from unsaturated ones through dehydrogenation. The wild-type expression levels of *OLE1* and the corresponding levels of oleic acid favor chlamydospore formation in *C. albicans* (Krishnamurthy et al., 2004). *NRG1* encodes one universal transcription inhibitor and regulates chlamydospore formation (Staib and Morschhäuser, 2005). However, the genes mentioned above had no significant differential expression levels or were not found in the transcriptome of *P. chrysosporium.* Therefore, the molecular mechanism of chlamydospore formation might vary in different fungi.

KEGG analyses illustrated 17 significantly changed genes enriched in seven pathways, which were involved in galactose metabolism; fatty acid degradation; ribosome, ascorbate, and aldarate metabolism; alpha-linolenic acid metabolism; starch and sucrose metabolism; and MAPK signaling pathway. Two major KEGG enrichment pathways involved in the change in the cell wall are the starch and sucrose metabolism and the Kss1 MAPK signaling pathway. β-Glucan has extensive distribution. It mainly exists in the cell walls of fungi and advanced plants (Barsanti et al., 2011) and has different chemical bonding modes compared with those of other sugars. β-1,3-Glucosidic bonds are the main chain in β-glucan, and β-1,6-glucosidic bonds as the branched chain are observed in yeast and fungi. In *Saprolegnia monoica*, which belongs to Oomycetes, high substrate concentrations of UDP–glucose in the membrane promote mycelial homogenate and microfibril production, and β-1,3-linked glucans are mainly produced; meanwhile, β-1,4-linked glucans are the only polysaccharide synthesized at low substrate concentrations in the presence of MgCl_2_ (Fèvre and Rougier, 1981). Starch and sucrose metabolism analysis showed that the expression of *UGP* was evidently downregulated when T72 was compared with CK72 and resulted in low UDP–glucose accumulation. The downregulation of *UGP* could promote sugar flux to produce 1,4-β-D-glucan, and downregulating *GLU* could reduce the hydrolysis of 1,4-β-D-glucan into β-D-glucose. The resulting 1,4-β-D-glucan accumulation was speculated as the major component of the thickened part of the cell wall in the chlamydospores. In compliance with this speculation, the enzymatic hydrolysis experiment revealed that the glucan content in the chlamydospore cell wall was higher than that in the hyphal cell wall. However, the cell wall organization may differ substantially between both structures, thereby masking polysaccharides that lie underneath and causing difficulty in accessing enzymes to it, resulting in more or less detected glucan/cellulose/chitin. Moreover, calcofluor white stained chitin and cellulose in the cell wall, but the contents of chitin and cellulose were not greater in the chlamydospores than in the hyphae via the enzymatic hydrolysis experiment. This finding also proved the complicated organization of the thickened cell wall. Therefore, additional experiments are needed to clarify this problem. Proteins are also found in the thick inner layer of the cell wall of *C. albicans* (Jansons and Nickerson, 1970), but these proteins were not detected in our study. MAPK cascade functions include induction of enzymes necessary for hyphal fusions by regulating different genes. Upregulation of transcription factors Tec1 in the MAPK signaling pathway might promote the extension of hypha and facilitate chlamydospore formation on it.

Although chlamydospore formation started at 36 h and peaked at 72 h, most transcriptional changes should occur earlier so that the cells have time to accommodate these changes and induce a corresponding result. Thus, a low number of DEGs were found between some conditions, and a more pronounced transcriptional response was observed at earlier time points.

Nitrogen limiting or starvation is a key factor triggering chlamydospore formation in *P. chrysosporium*. Further hyphal extension would be inhibited during the transportation of nutrients along the long vegetative hyphae. In this circumstance, the hyphae would grow because of the carbon sources stored in the chlamydospore structure rather than because of long-distance transportation. The fungal cell wall is made of polysaccharides and can be stored as energy; thus, the formed chlamydospores for energy storage could supply a stable energy for germination. This process could be completed by controlling the transfer and decomposition of stored nutrients.

## Conclusion

The cell wall of the chlamydospores was thicker than that of the hyphae, and more β-glucans were exposed to β-glucanase in the chlamydospores than in the hyphae by enzymatic hydrolysis. Thus, the cell wall structure and organization of the chlamydospores and the hyphae significantly differed. Chlamydospore formation in *P. chrysosporium* was characterized using RNA-seq. The results provided a basic understanding of the molecular mechanisms under this process, including MAPK, phosphatases, and transcription factors. The chlamydospore formation in P. chrysosporium was encoded by a large number of genes subject to strict patterns of temporal regulation. These comprehensive analyses provide molecular evidence that could promote the understanding of morphological variation in chlamydospore formation and serve as a potential blue print for future research on this process in basidiomycetes and other fungi. The genes mentioned in this process will be tested via gene knockout in our future work.

## Data Availability Statement

The datasets generated for this study can be found in the SRA, SRP153122.

## Author Contributions

LL, HL, and YYL performed the experiments and wrote the main text of the manuscript. HW designed and directed the experiments. YL analyzed the data. All authors have read and approved the manuscript.

## Conflict of Interest

The authors declare that the research was conducted in the absence of any commercial or financial relationships that could be construed as a potential conflict of interest.

## Footnotes

1 https://www.ncbi.nlm.nih.gov/sra
