# Supplementary figures and images for: Whole Transcriptome Analysis Provides Insights Into the Molecular Mechanisms of Chlamydospore-Like Cell Formation in Phanerochaete chrysosporium
Source: Front Microbiol. 2020 Dec 7;11:527389. doi: 10.3389/fmicb.2020.527389 (PMC7750433; doi:10.3389/fmicb.2020.527389)

# Pearson correlation between samples

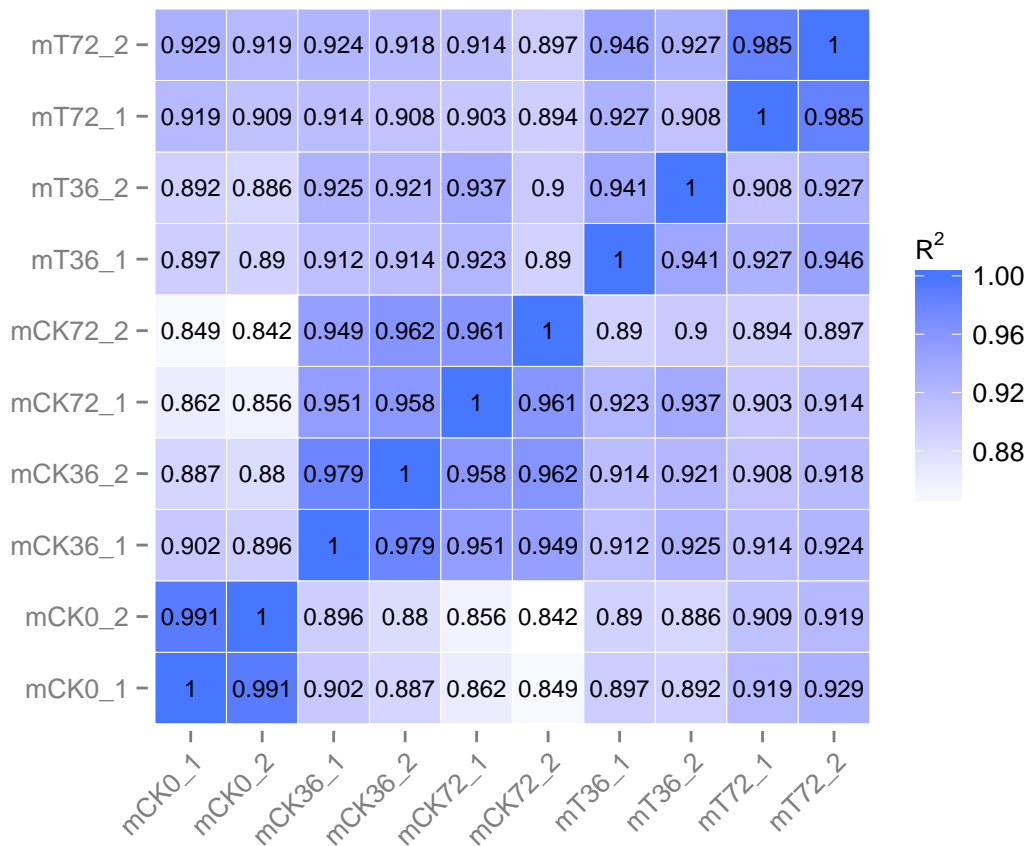

Supplement: Supplementary file 1 [file Data_Sheet_1.ZIP › Correlation/cor_pearson.pdf]

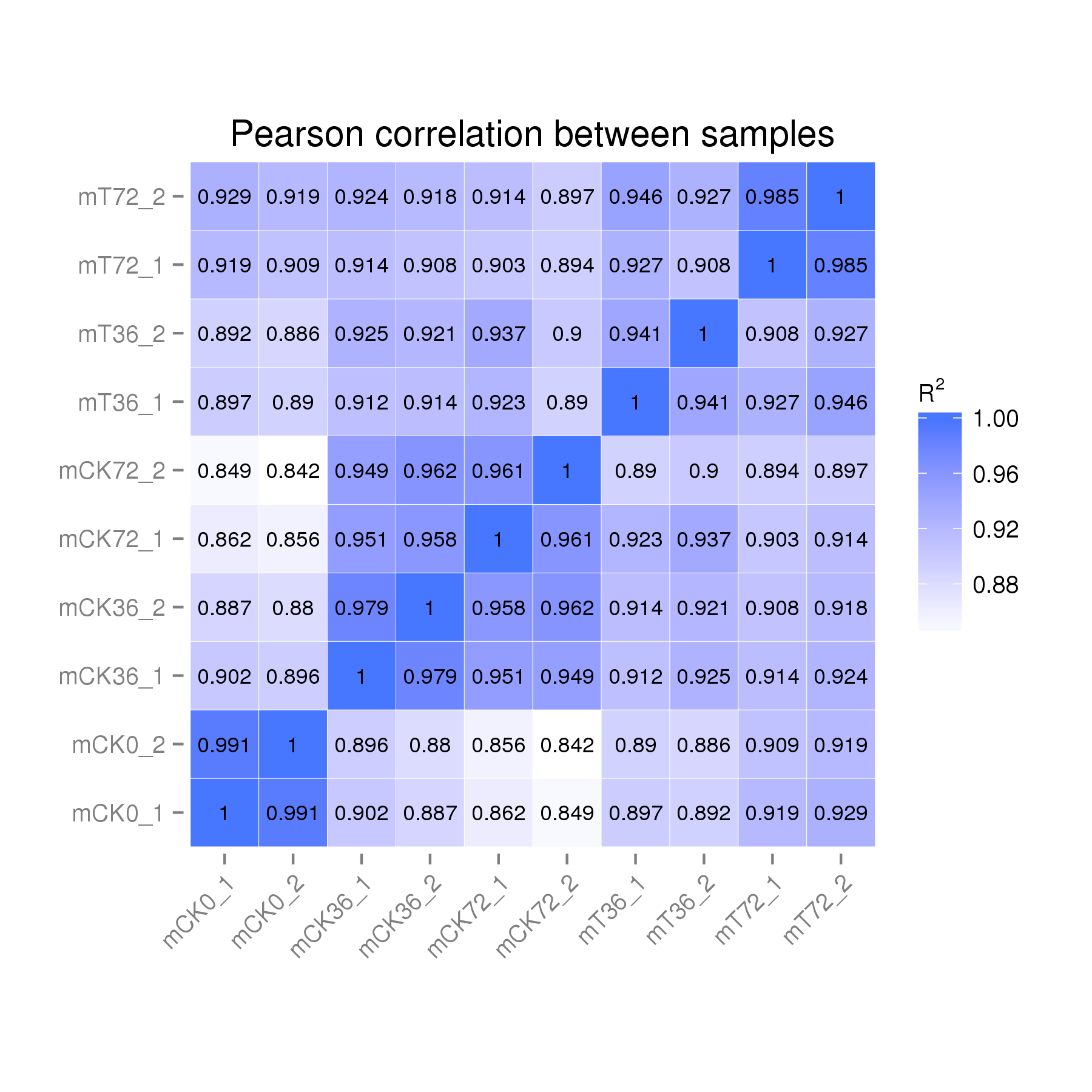

Supplement: Supplementary file 1 [file Data_Sheet_1.ZIP › Correlation/cor_pearson.png]

mCK0\_1 vs mCK0\_2

$R^2 = 0.991$

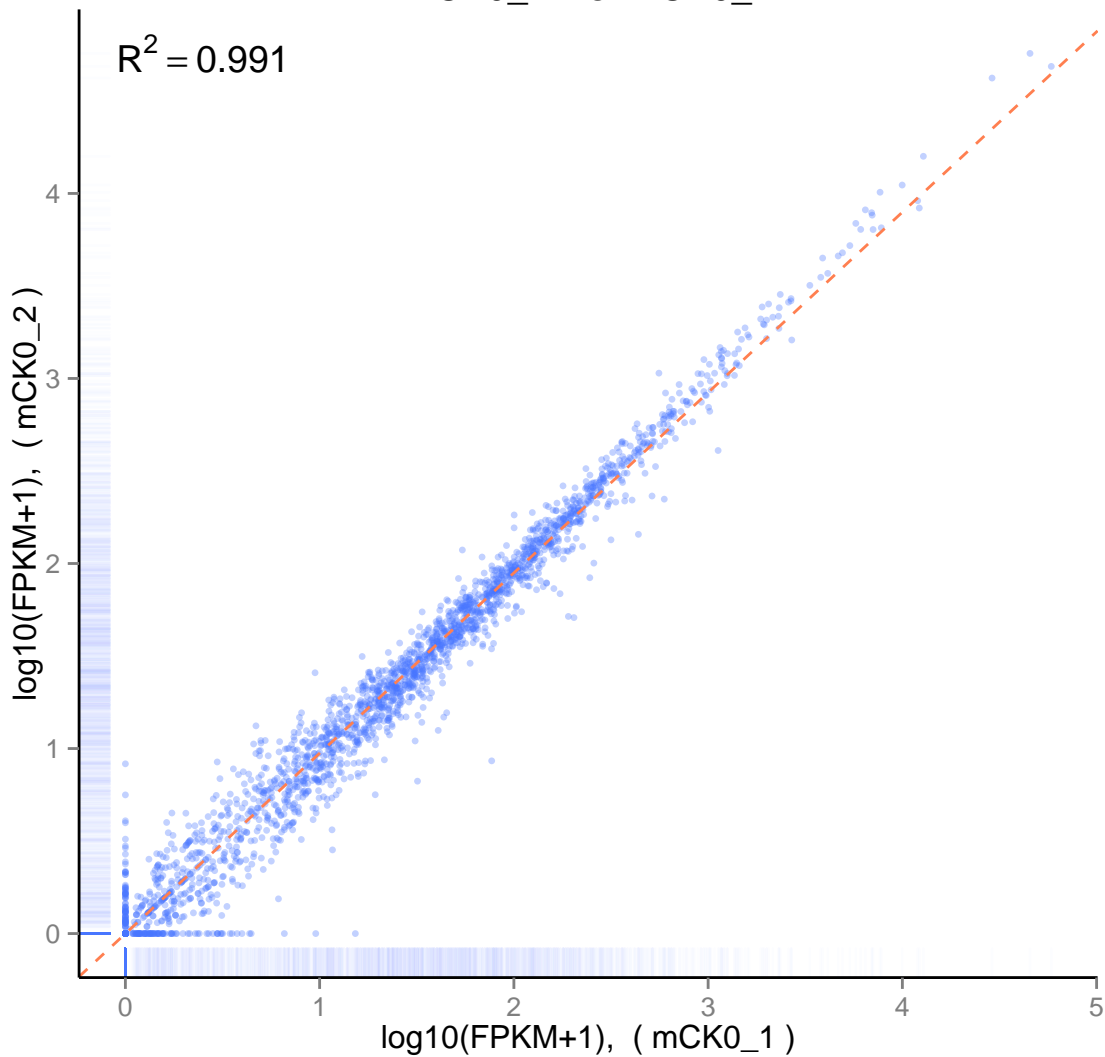

Supplement: Supplementary file 1 [file Data_Sheet_1.ZIP › Correlation/mCK0_1_vs_mCK0_2.scatter.pdf]

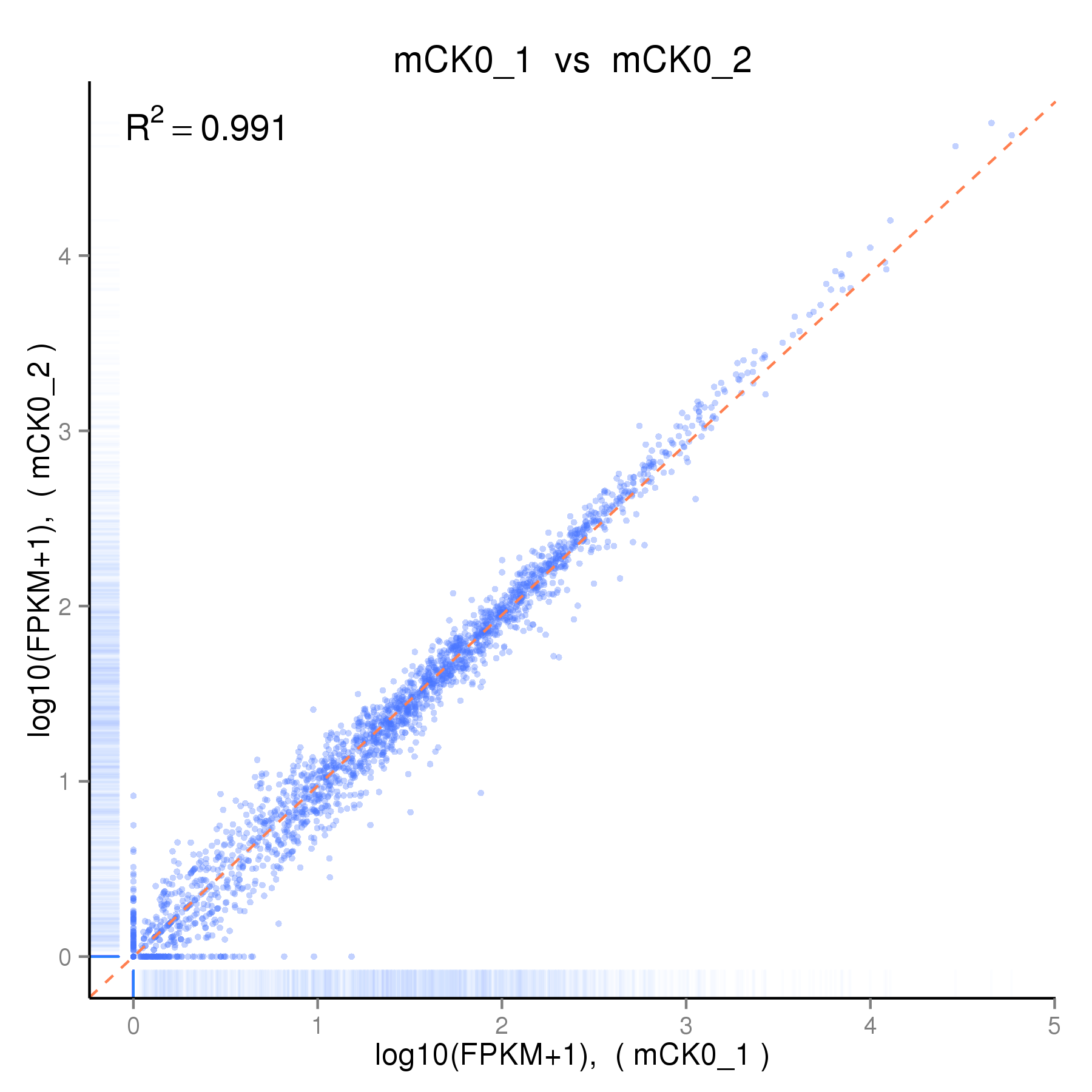

Supplement: Supplementary file 1 [file Data_Sheet_1.ZIP › Correlation/mCK0_1_vs_mCK0_2.scatter.png]

mCK36\_1 vs mCK36\_2

$R^2 = 0.979$

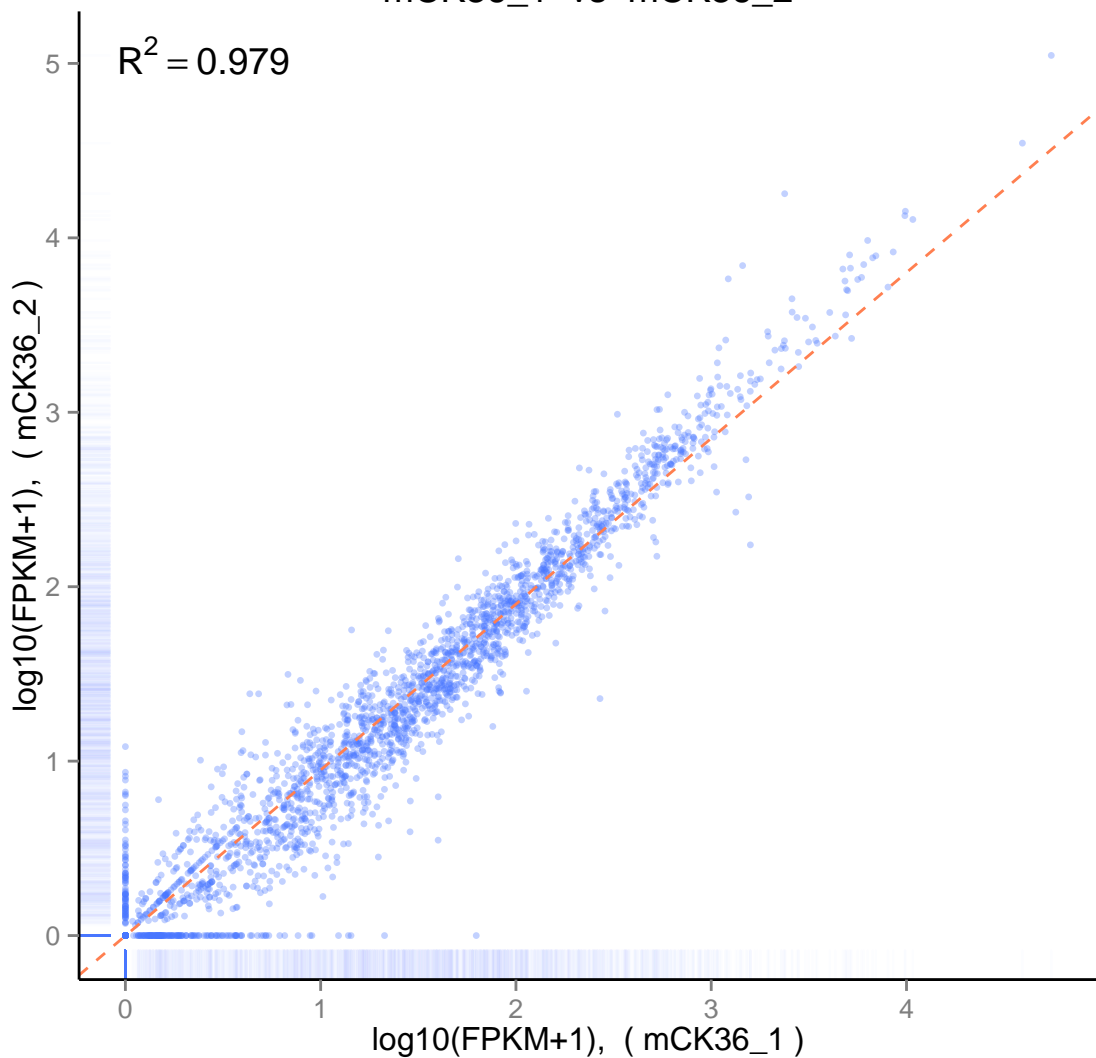

Supplement: Supplementary file 1 [file Data_Sheet_1.ZIP › Correlation/mCK36_1_vs_mCK36_2.scatter.pdf]

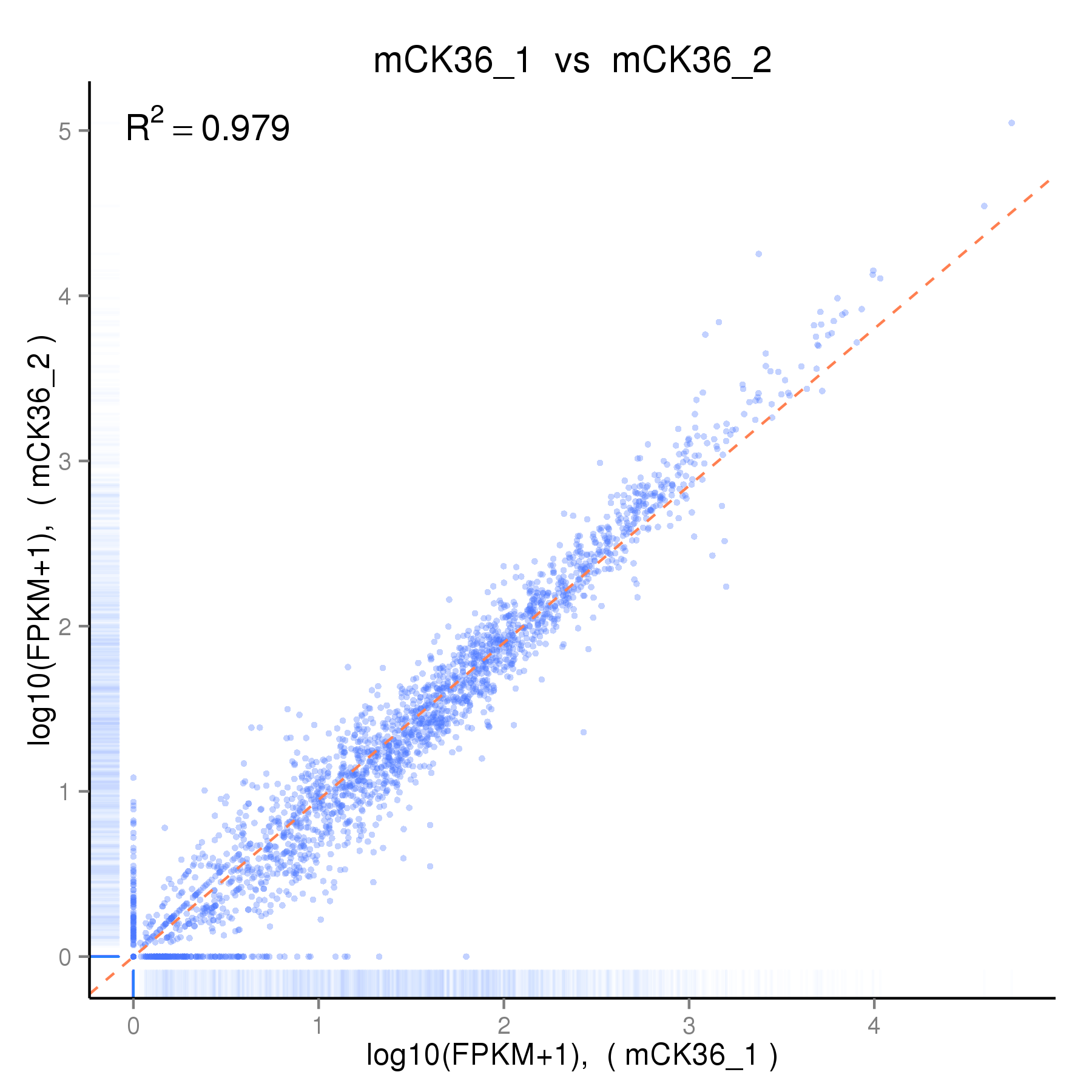

Supplement: Supplementary file 1 [file Data_Sheet_1.ZIP › Correlation/mCK36_1_vs_mCK36_2.scatter.png]

mCK72\_1 vs mCK72\_2

$R^2 = 0.961$

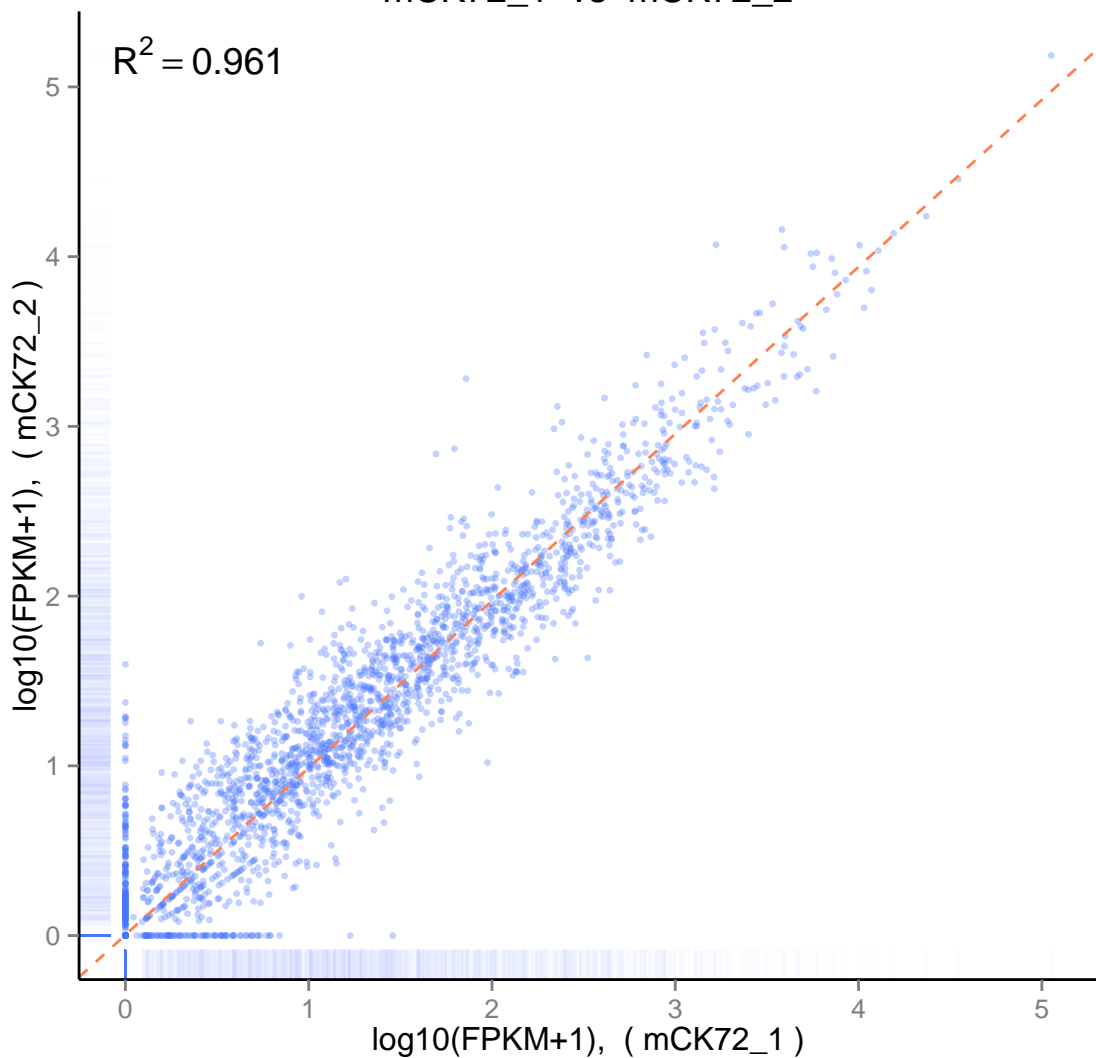

Supplement: Supplementary file 1 [file Data_Sheet_1.ZIP › Correlation/mCK72_1_vs_mCK72_2.scatter.pdf]

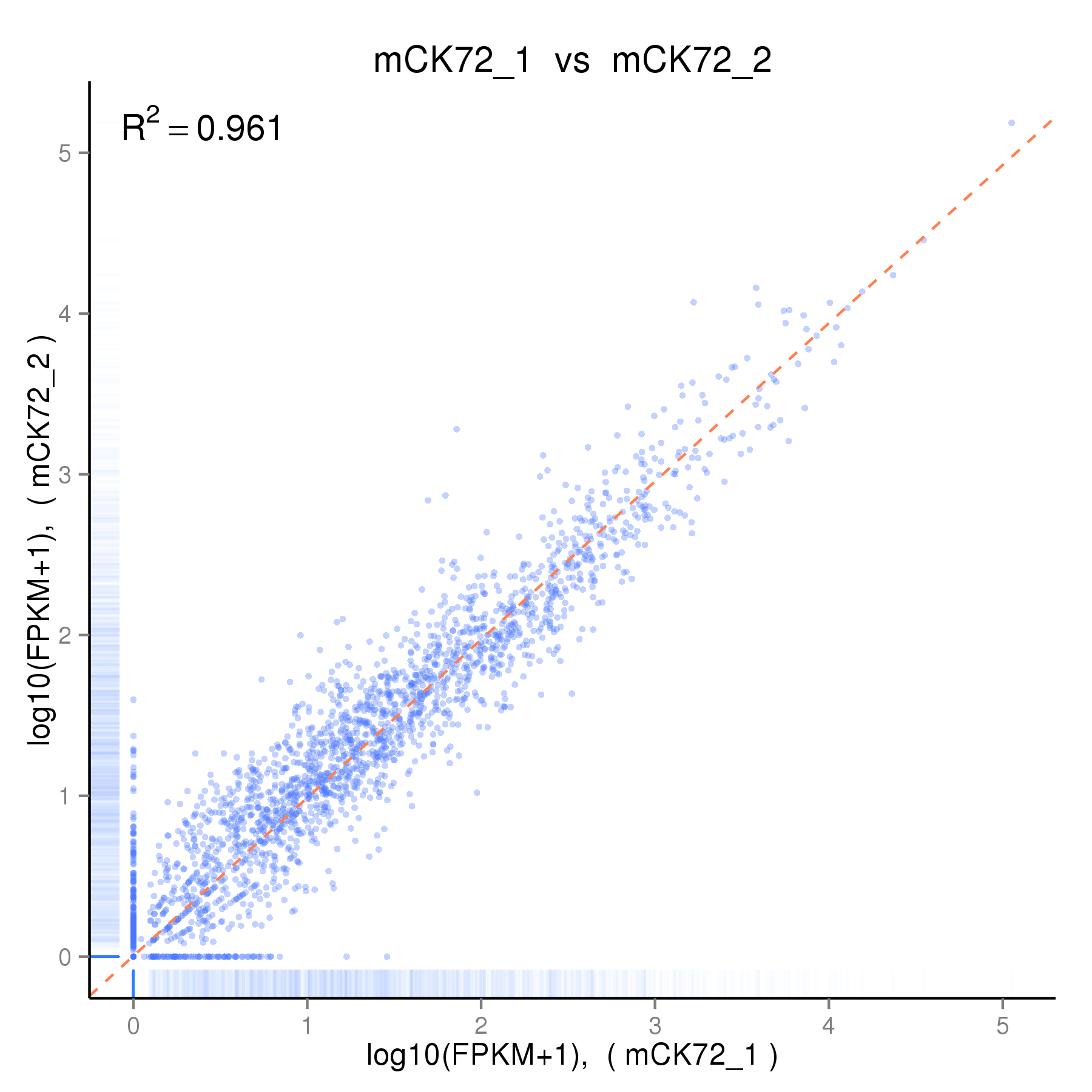

Supplement: Supplementary file 1 [file Data_Sheet_1.ZIP › Correlation/mCK72_1_vs_mCK72_2.scatter.png]

mT36\_1 vs mT36\_2

$R^2 = 0.941$

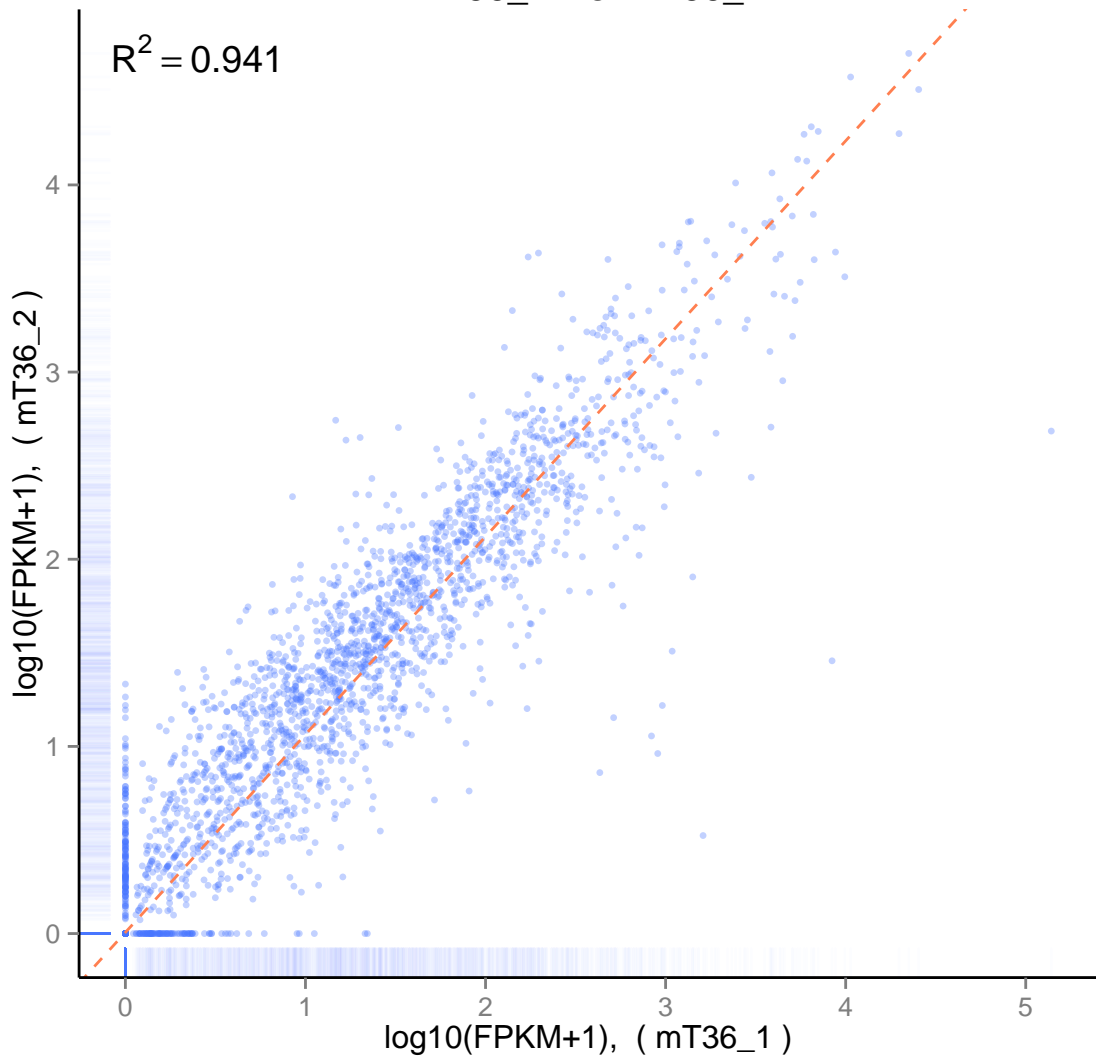

Supplement: Supplementary file 1 [file Data_Sheet_1.ZIP › Correlation/mT36_1_vs_mT36_2.scatter.pdf]

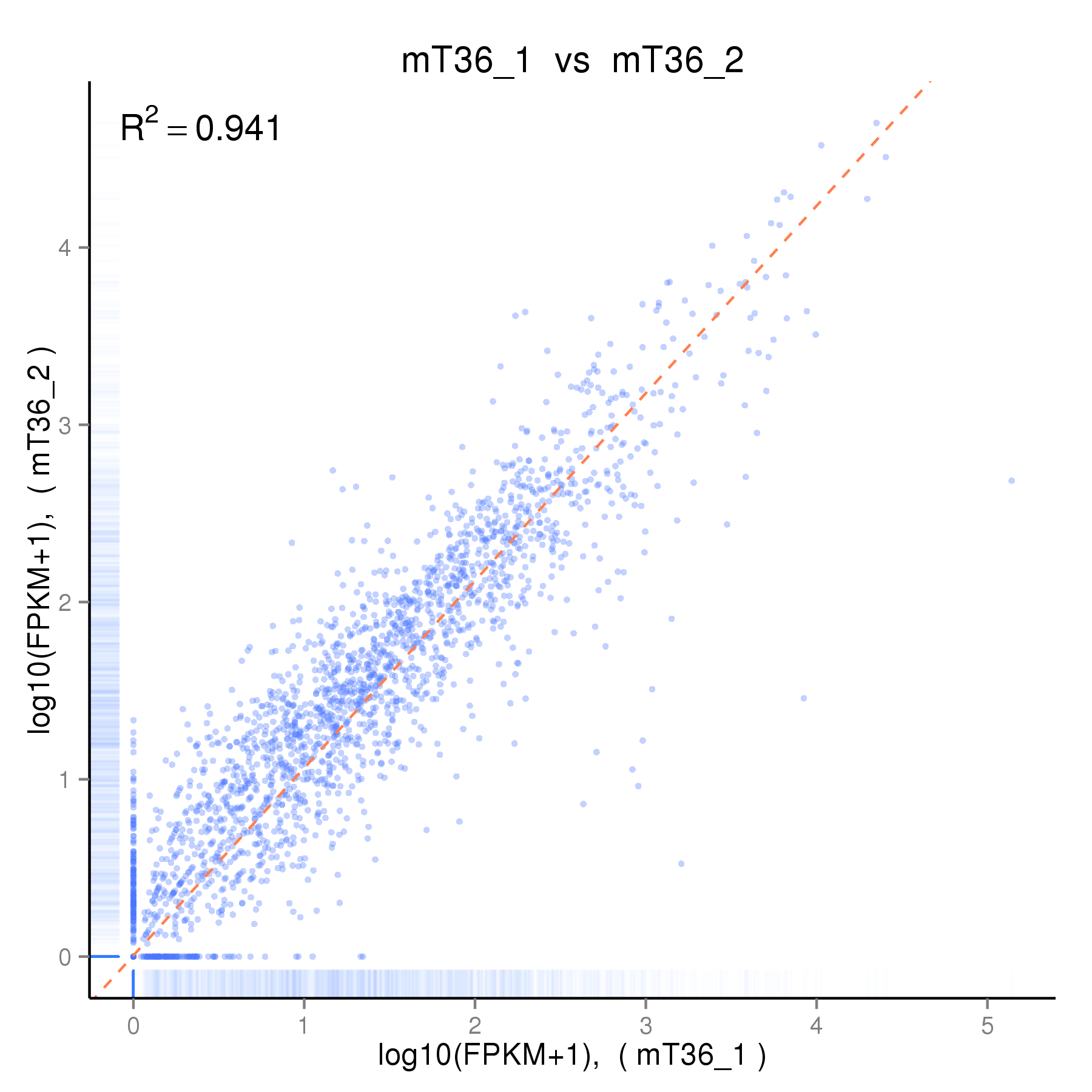

Supplement: Supplementary file 1 [file Data_Sheet_1.ZIP › Correlation/mT36_1_vs_mT36_2.scatter.png]

mT72\_1 vs mT72\_2

$R^2 = 0.985$

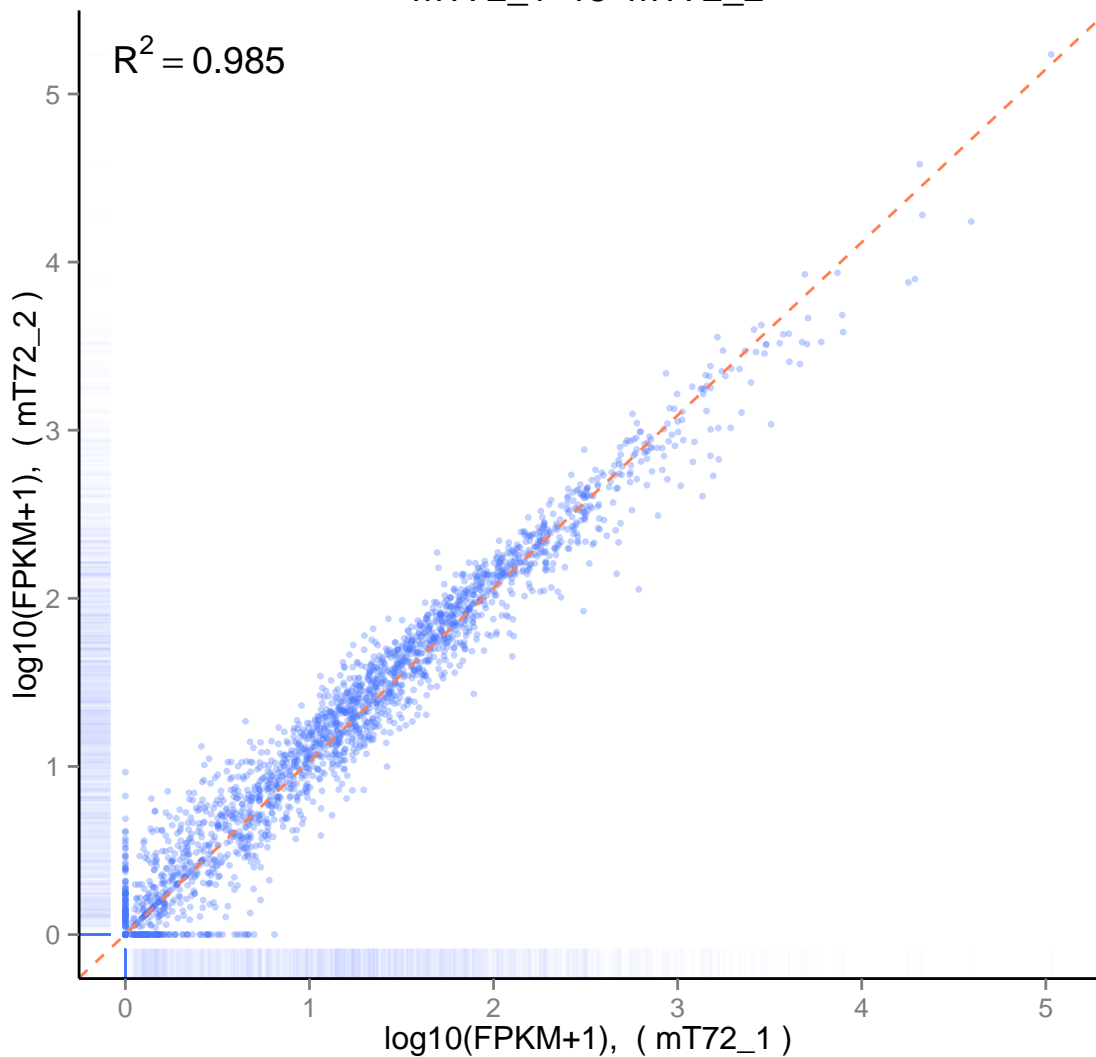

Supplement: Supplementary file 1 [file Data_Sheet_1.ZIP › Correlation/mT72_1_vs_mT72_2.scatter.pdf]

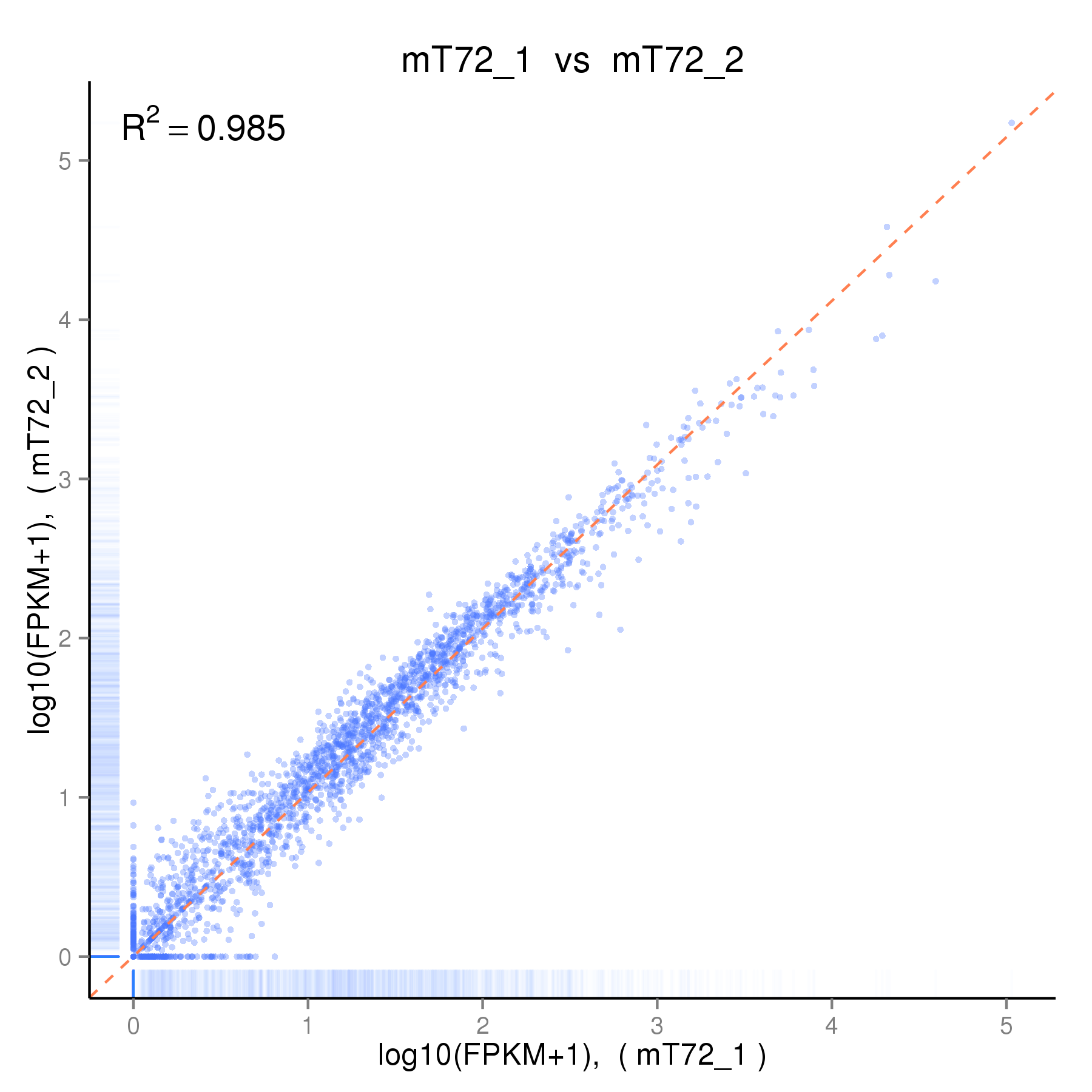

Supplement: Supplementary file 1 [file Data_Sheet_1.ZIP › Correlation/mT72_1_vs_mT72_2.scatter.png]
