# Supplementary material for: Whole Transcriptome Analysis Provides Insights Into the Molecular Mechanisms of Chlamydospore-Like Cell Formation in Phanerochaete chrysosporium
Source: Front Microbiol. 2020 Dec 7;11:527389. doi: 10.3389/fmicb.2020.527389 (PMC7750433; doi:10.3389/fmicb.2020.527389)
Supplement: Supplementary file 3 [file Table_1.DOCX]

Supplementary Material

# Supplementary Figures and Tables

## Supplementary Figures


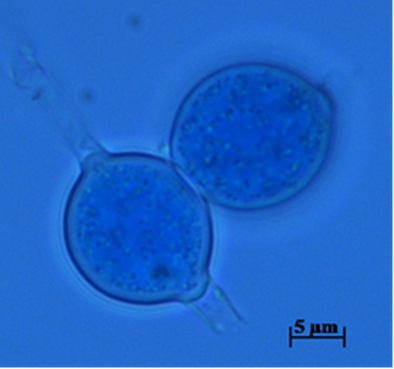


**Supplementary Figure S1.** Coomassie brilliant blue staining of chlamydospore.

## Supplementary Tables

Supplementary Table S1. Primers used in indicating the reliability and accuracy of the RNA-seq data

| Gene | Primer sequences (5’-3’) |
| --- | --- |
| *CHI-F* | CGCAAGGGTCCTCCAATACAATG |
| *CHI-R* | GCGATGACGTTGAGGCGATAGAT |
| *TEC 1-F* | AAGCGGACACCGAAGCAG |
| *TEC 1-R* | GCCGCCGTAGTACCAGAAGTTC |
| *SLT 2-F* | GACAGGGCTACCATTCGTTGA |
| *SLT 2-R* | ATGGGAGAAGTGACGCAAGCG |
| *FKS 2-F* | GGATTCGCCGTGACAAGGATT |
| *FKS 2-R* | GGACGGAGACCCAGAAGTAGGTC |
| *HOG 1-F* | TCTCCACCGTCTCCTCACCTC |
| *HOG 1-R* | ATTGCTGGGTTTCAGGTCTCG |
| *GLU-F* | CCAGCGAGAGGACACTGAGAGA |
| *GLU-R* | ATCCAGCAACCAATCGTTCTCC |
| *Actin-F* | GTGGTGTCTCATCCGTATTCCTT |
| *Actin-R* | TTCGTGATATGCGAGTTCTGGT |
